# Proline Hydroxylation in Cell Wall Proteins: Is It Yet Possible to Define Rules?

**DOI:** 10.3389/fpls.2017.01802

**Published:** 2017-10-17

**Authors:** Harold Duruflé, Vincent Hervé, Thierry Balliau, Michel Zivy, Christophe Dunand, Elisabeth Jamet

**Affiliations:** ^1^Laboratoire de Recherche en Sciences Végétales, Université de Toulouse, CNRS, UPS, Toulouse, France; ^2^INRS – Institut Armand Frappier, Laval, Canada; ^3^PAPPSO, GQE Le Moulon, INRA, Univ. Paris-Sud, CNRS, AgroParisTech, Université Paris-Saclay, Gif-sur-Yvette, France

**Keywords:** *Arabidopsis thaliana*, cell wall protein, hydroxyproline, mass spectrometry, proline hydroxylation, proline-rich protein, post-translational modification

## Abstract

Cell wall proteins (CWPs) play critical and dynamic roles in plant cell walls by contributing to developmental processes and response to environmental cues. Since the CWPs go through the secretion pathway, most of them undergo post-translational modifications (PTMs) which can modify their biological activity. Glycosylation is one of the major PTMs of CWPs and refers to *N*-glycosylation, *O*-glycosylation and glypiation. Each of these PTMs occurs in different amino acid contexts which are not all well defined. This article deals with the hydroxylation of Pro residues which is a prerequisite for *O*-glycosylation of CWPs on hydroxyproline (Hyp) residues. The location of Hyp residues is well described in several structural CWPs, but yet rarely described in other CWPs. In this article, it is studied in detail in five *Arabidopsis thaliana* proteins using mass spectrometry data: one of them (At4g38770, AtPRP4) is a structural CWP containing 32.5% of Pro residues arranged in typical motifs, the others are either rich (27–28%, At1g31580 and At2g10940) or poor (6–8%, At1g09750 and At3g08030) in Pro residues. The known rules of Pro hydroxylation allowed a good prediction of Hyp location in AtPRP4. However, they could not be applied to the other proteins whatever their Pro content. In addition, variability of the Pro hydroxylation patterns was observed within some amino acid motifs in all the proteins and new patterns of Pro hydroxylation are described. Altogether, this work shows that Hyp residues are present in more protein families than initially described, and that Pro hydroxylation patterns could be different in each of them. This work paves the way for completing the existing Pro hydroxylation code.

## Introduction

Cell wall proteins (CWPs) are important players in plant cell walls, otherwise mainly constituted of polysaccharides, and eventually of phenolic compounds around differentiated lignified cells (Carpita and Gibeaut, 1993). They have been involved in the remodeling of cell wall polymers networks by hydrolysing covalent bounds, inserting newly synthesized polysaccharides, cross-linking together structural proteins, proteins and polysaccharides or polysaccharides and phenolic compounds (Franková and Fry, 2013; Cosgrove, 2015). Together with extracellular peptides, some CWPs have also been involved in signaling, thus allowing cell-to-cell communication (Matsubayashi and Sakagami, 2006; Lamport and Várnai, 2013; Tavormina et al., 2015). Altogether, CWPs and peptides contribute to both developmental processes and response to environmental cues (Tenhaken, 2015; Borassi et al., 2016). Most of them undergo post-translational modifications (PTMs) during their transport through the secretion pathway (Faye et al., 2005; Kim and Brandizzi, 2016) which can modify their conformation, their biological activity and/or their ability to interact with cell wall components (Lannoo and Van Damme, 2015; Baer and Millar, 2016; Strasser, 2016). As an example, the site-directed mutagenesis of the *N*-glycosylation motifs of a class III peroxidase was shown to reduce its thermal stability, its catalytic activity and to modify its conformation (Lige et al., 2001).

During recent years, proteomics has facilitated a better knowledge of the plant cell wall proteome by increasing its coverage thanks to the design of specific strategies able to recover protein extracts enriched in extracellular proteins from organs of several model plants and crops (Lee et al., 2004; Albenne et al., 2013; Komatsu and Yanagawa, 2013; Rodríguez-Celma et al., 2016). Beyond the identification of proteins, technological advances have also permitted description of their PTMs. Various methods have been developed to address this particular question. In particular, immobilized affinity chromatography (IMAC) and lectin-affinity chromatography have allowed studying protein phosphorylation and glycosylation, respectively (Ytterberg and Jensen, 2010; Nakagami et al., 2012; Ruiz-May et al., 2014; Canut et al., 2016).

Glycosylation is one of the major PTMs of CWPs and refers to *N*-glycosylation, *O*-glycosylation and addition of glycophosphatidylinositol (GPI)-anchors, also named glypiation (Faye et al., 2005). Each of these PTMs occurs on specific amino acid sequences. *N*-glycosylation is the best described. It occurs on Asn residues in Asn-X-Ser/Thr motifs, where X cannot be a Pro residue. In these motifs, the hydroxyl functional group of Ser/Thr residues was shown to be required in the transglycosylation reaction on the Asn residue (Bause and Legler, 1981), whereas the presence of a Pro residue modifies the local conformation of the protein, thus preventing its *N*-glycosylation (Bause, 1983). The different structures of *N*-glycans are well-known, thus allowing systematic search in mass spectrometry (MS) data obtained in conditions preserving glycan-peptide bonds (Ruiz-May et al., 2012). GPI-anchors are transferred by a transamidase to a carboxy-terminal GPI-attachment signal peptide which can be predicted by bioinformatics (Eisenhaber et al., 2003). Several targeted proteomic studies have contributed to the identification of GPI-anchored proteins and some of them could be released from plasma membrane fractions by a phospholipases C or D which cleave GPI-anchors (Borner et al., 2003; Elortza et al., 2003; Elortza et al., 2005). *O*-glycosylation is the most complex type of glycosylation. In plant CWPs, it can occur on Ser and hydroxyproline (Hyp) residues (Faye et al., 2005). Galactose can be linked to Ser and Hyp residues whereas arabinose can only be linked to Hyp residues (Canut et al., 2016). According to the so-called Hyp contiguity hypothesis initially proposed for hydroxyproline-rich proteins (HRGPs), contiguous Hyp residues are arabinosylated and clustered non-contiguous Hyp residues are galactosylated (Shpak et al., 2001). Then, glycosyltransferases can extend the *O*-glycans in different ways depending on the initial pattern of Pro hydroxylation. The correct *O*-glycosylation of HRGPs was shown to be required for their conformation or their biological activity (Stafstrom and Staehelin, 1986; Velasquez et al., 2011).

**FIGURE 1:**
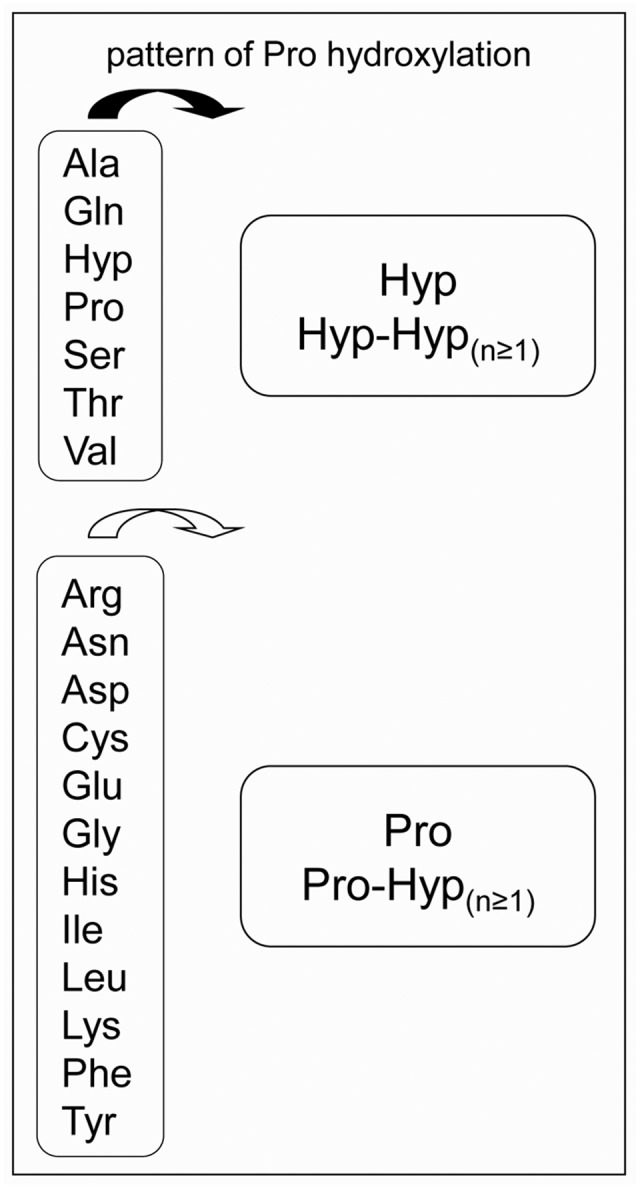
The proposed Pro-hydroxylation code for plant cell wall proteins. On the left side, amino acids preceding Pro residues. On the right side, patterns of Pro hydroxylation (Canut et al., 2016).

Pro hydroxylation is a major step for *O*-glycosylation, but it is still difficult to predict in which amino acid context it occurs. In a previous review, we have proposed an extended Pro hydroxylation code (Canut et al., 2016), based on both (i) the initial Pro hydroxylation code (Kieliszewski and Lamport, 1994) and (ii) additional experimental LC-MS/MS and Edman sequencing data. The extended code has taken into account more protein and peptide families than the former one, including structural proteins like HRGPs and Pro/Hyp-rich proteins, solanaceous lectins, allergens, systemins and CLE peptides. Briefly, Pro residues could be hydroxylated when they are located after Ala, Gln, Hyp, Pro, Ser, Thr, and Val residues, whereas the first Pro residue following the other amino acids could not be hydroxylated (**Figure 1**). Only little information is available regarding Trp and Met residues. In a large mutagenesis screen performed on the amino acids surrounding the only Pro residue of sporamin shown to be hydroxylated, Trp and Met were not shown to favor the hydroxylation of the following Pro residue (Shimizu et al., 2005).

In this article, our aim was to test the extended Pro hydroxylation code on a new set of CWPs including non-structural CWPs. We have thus selected five CWPs with various contents in Pro residues. Three of them were rich in Pro residues among which AtPRP4 which is a structural CWP (Fowler et al., 1999) and two of them were poor in Pro residues. We have performed a deep data mining on two recent cell wall proteomic studies performed on rosettes and stems of *Arabidopsis thaliana* (Hervé et al., 2016; Duruflé et al., 2017). From the fine analysis of MS data, we have compared the observed patterns of Pro/Hyp location to the predicted ones according to the Pro hydroxylation extended code. The limits of the existing extended Pro hydroxylation code are discussed and new motifs are described.

## Results

### Mapping of Hyp Residues

For this analysis, we have taken advantage of two cell wall proteomics studies which have lead to the identification of numerous CWPs, 361 in rosettes and 302 in stems, i.e., 397 different CWPs (Hervé et al., 2016; Duruflé et al., 2017). This body of data corresponded to three independent experiments (two for rosettes and one for stems), each of them including three biological replicates. The parameters used for peptide identification included a possible mass delta of 15.99 Da for each Pro residue, corresponding to its hydroxylation. As an example, in one of the rosette experiment, 79% of the identified CWPs were predicted to be *N*-glycosylated (presence of the PS00001 PROSITE motif), whereas 17.5% had at least one peptide carrying a Hyp residue. Among the latters and in addition to the proteins described below, there were lectins, Asp proteases, lipases acylhydrolases of the GDSL family, and class III peroxidases.

Five CWPs were selected on the basis of the following criteria: (i) their abundance in these aerial organs, as shown by the high number of sequenced peptides for each of them (from 89 to 533, depending on the protein); and (ii) a high sequence coverage (from 26 to 72% of the mature protein) (**Table 1**). The MS data corresponding to these five proteins are given in Supplementary Table S1. In addition, none of them has already been shown to contain Hyp residues. At4g38770 (AtPRP4) is a Pro-rich protein and its gene was shown to be expressed in aerial organs (Fowler et al., 1999). At1g09750 is a predicted Asp protease. At1g31580 (ECS1/CXc750) was assumed to be involved in resistance mechanisms (Aufsatz et al., 1998). At3g08030 (AthA2-1) has a predicted DUF642 domain (Vázquez-Lobo et al., 2012). Finally, At2g10940 is a protein showing homology to non-specific plant lipid transfer proteins. Three out of these CWPs have amino acid sequences rich in Pro residues as calculated from their mature sequence: AtPRP4 (32.5% Pro), At1g31580 (27.9%), and At2g10940 (27.3%). The two others, At1g09750 and At3g08030, are poor in Pro residues (7.3 and 6.4%, respectively). Contrarily to the three former proteins which exhibit Pro-rich motifs, the latter ones have dispersed Pro residues.

**Table 1:** Sequence coverage and number of peptides analyzed by LC-MS/MS for each protein.

	Size of the protein (number of amino acids)	Average percentage of sequence coverage	Total number of sequenced peptides
At4g38770 (AtPRP4)	419	51	131
At1g09750	428	26	450
At1g31580	68	60	89
At3g08030	353	72	533
At2g10940	264	60	250

*The list of the sequenced peptides is provided in Supplementary Table S1. The percentage of sequence coverage is calculated for the mature protein sequence and takes the results of the three experiments into account.*

**FIGURE 2:**
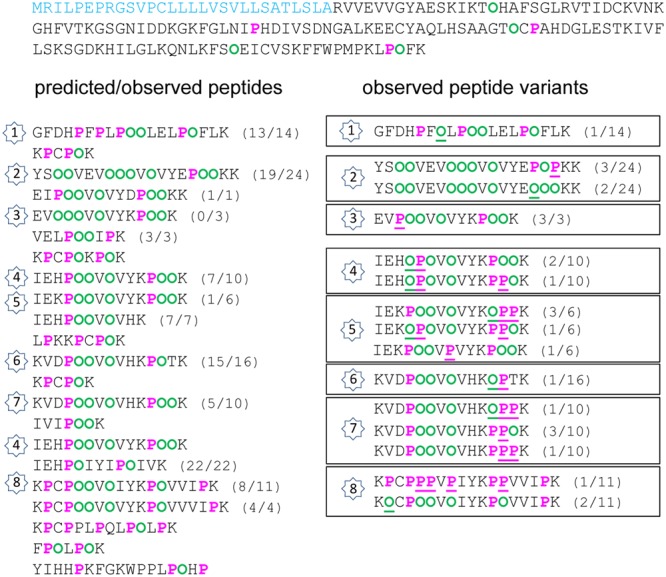
Hydroxylation of Pro residues in the amino acid sequence of At4g38770 (AtPRP4) encoding a Pro-rich protein. The amino acid sequence of AtPRP4 is written from left to right and from top to bottom. The predicted peptide signal is indicated in light blue. The Pro-rich domain is displayed in order to emphasize repetitive sequences and tryptic peptides (one per line). On the left side, predicted Pro (P) and Hyp (O) residues are in pink and green, respectively. On the right side, observed Pro and Hyp residues at unexpected positions are underlined. For each peptide, the number between brackets corresponds to its frequency of occurrence, expressed as a ratio between the number of observed peptides and the total number of sequenced peptides. The numbers inside stars allow the comparison between the predicted/observed peptides (on the left side) and the corresponding observed Pro/Hyp peptide variants (on the right side).

**FIGURE 3:**
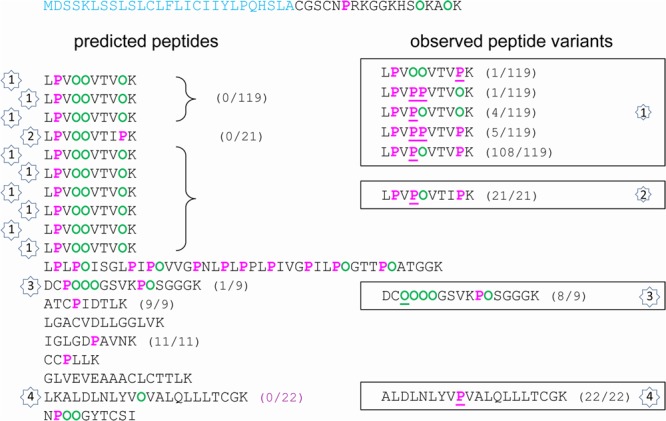
Hydroxylation of Pro residues in the amino acid sequence of At2g10940 encoding a protein homologous to non-specific lipid transfer protein. The amino acid sequence of At2g10940 is written from left to right and from top to bottom. The predicted peptide signal is indicated in light blue. The Pro-rich domain is displayed in order to emphasize repetitive sequences and tryptic peptides (one per line). On the left side, predicted Pro (P) and Hyp (O) residues are in pink and green, respectively. On the right side, observed Pro and Hyp residues at unexpected positions are underlined. For each peptide, the number between brackets corresponds to its frequency of occurrence, expressed as a ratio between the number of observed peptides and the total number of sequenced peptides. The numbers inside stars allow the comparison between the predicted/observed peptides (on the left side) and the corresponding observed Pro/Hyp peptide variants (on the right side).

The extended Pro hydroxylation code was applied to predict the location of Hyp residues in the five amino acid sequences and to compare them to the observed ones. All details are given in Supplementary Figure S1, and simplified views of AtPRP4 and At2g10940 are shown in **Figures 2** and **3**. The sequences including the predicted Hyp residues are shown on the left and the observed ones are framed on the right of each figure. No obvious difference could be found between the three datasets regarding the frequency of occurrence of the different peptide variants according to the location of Pro and Hyp residues (Pro/Hyp peptide variants). In particular, no difference could be found between the rosette and the stem samples. The MS/MS data were manually checked as shown in Supplementary Figure S2 for two peptides of AtPRP4. All the Pro/Hyp locations were confirmed with the exception of two motifs in the amino acid sequence of At1g09750 (GPM and LPM). We could not discriminate between the hydroxylation of a Pro residue and the oxidation of a Met residue (Supplementary Table S2B and Figure S1). Thus, we did not retain the hypothesis of a Pro hydroxylation in these motifs in the following.

Several observations could be done. (i) A very high proportion of Pro residues was hydroxylated in the three Pro-rich proteins (69/92 in AtPRP4, 8/10 in At1g31580, 36/49 in At2g10940). (ii) Only a few Hyp residues could be found in proteins poor in Pro residues, and rarely at predicted positions (none in At1g09750; only at two out of nine predicted possibilities, and two at unexpected positions, but at a very low frequency, in At3g08030). (iii) For a given peptide, several variants could be observed. For example, two variants of GFDHPFPLPPPLELPPFLK and three variants of YSPPVEVPPPVPVYEPPPKK were found in AtPRP4: GFDHPF**P**LPOOLELPOFLK as predicted, and GFDHPF**O**LPOOLELPOFLK; YSOOVEVOOOVOVYE**POO**KK as predicted, YSOOVEVOOOVOVYE**POP**KK, and YSOOVEVOOOVOVYE**OOO**KK (Supplementary Figure S2). (iv) The observed discrepancies between the predicted and the observed Pro/Hyp locations could be either a Pro instead of a Hyp residue or vice-versa. (v) Some discrepancies could be systematically observed, as the F**O**OR motif in At1g31580 instead of the predicted F**P**OR motif.

This survey has allowed the fine mapping of Pro/Hyp residues in the five selected CWPs. The next issue was to know how efficiently the extended Pro hydroxylation code could predict their location.

### Efficiency of the Prediction of the Location of the Pro/Hyp Residues

For each protein sequence, the total number of Pro and Hyp positions was recorded and compared to the number of correct predictions (**Table 2** and Supplementary Table S2). The percentage of mis-predictions was found to range from 5.1 to 46.7%. The best prediction was obtained for AtPRP4 which is a HRGP, i.e., a canonical protein with regard to the proposed rule. Except one motif in peptide 3 (**Figure 2** and Supplementary Figure S1A), V**P**OOV instead of the predicted V**O**OOV, all the other predicted motifs were found at least once. Some variability was observed within 12 motifs located in seven peptides (numbered 1, 2, and 4–8 on **Figure 2**). The KPPPK motif was the most variable one, with the following variants: KPOOK as predicted (peptides 3–5 and 7), KP**P**OK (peptides 4, 5, and 7), K**OPP**K (peptides 5 and 7), and KP**PP**K (peptide 7). Other motifs including three Pro/Hyp residues were also variable, such as EPPPK in peptide 2 (EPOOK as predicted, EPO**P**K and E**O**OOK), HPPPV in peptide 4 (HPOOV as predicted and H**OP**O), CPPPV in peptide 8 (CPOOV as predicted and CP**PP**V). The other cases of variability concerned shorter motifs such as FPL (F**O**L in peptide 1), VPV/I (V**P**V in peptide 5 and V**P**I in peptide 8), KPPT/V (K**O**PT in peptide 6, KP**P**V in peptide 8). However, all these Pro/Hyp peptide variants were not the prevailing forms of the motifs (see Supplementary Table S2).

**Table 2:** Efficiency of the prediction of Pro/Hyp location in CWP amino acid sequences according to the proposed rules.

Accession number	Number of analyzed Pro/Hyp positions	Number of unexpected location of Pro/Hyp residues	Percentage of mis-predictions
At4g38770	868	44	5.1
At1g09750	343	104	30.3
At1g31580	439	154	35.1
At3g08030	502	225	44.8
At2g10940	602	281	46.7

*Five CWP sequences were analyzed by LC-MS/MS (Supplementary Table S1) and the percentage of mis-predictions was calculated (Supplementary Table S2).*

The prediction of Pro and Hyp location was much less efficient for the four other proteins, irrespectively, to their Pro content. Regarding the two proteins with a low Pro content, the percentage of mis-prediction of Pro/Hyp location was very high (30.3% for At1g09750 and 44.8% for At3g08030). Hyp was only found in At3g08030, but in solely three motifs (VOF, G**O**H, and L**O**L) and at a low frequency (**Table 3**). Regarding the two proteins with a high percentage of Pro residues (At1g31580 and At2g10940), the situation was very different. Although they both exhibited a high percentage of Pro residues, the proposed rules did not allow reaching a high level of correct prediction of Pro/Hyp location. For At1g31580, peptide variants could mostly be observed for short motifs like RPI/R/T in peptides 1 and 3 (RPI/R/T as predicted and R**O**I/R/T as observed) and VPI/G in peptides 1 and 2 (VOI/G as predicted and V**P**I/G) (Supplementary Figure S2C). Two larger motifs were variable: FPPR in peptide 2 (FPOR as predicted and F**O**OR); LPPY in peptide 3 (LPOY as predicted and LP**P**Y). With the exception of the FPPR motif in which Pro residues were always both hydroxylated and the VPI motif which was found in the VOI form in all but three cases out of 55, all the other variants were found in the one third/two third proportion between predicted and mis-predicted variants or vice versa. For At2g10940, variability was observed in four motifs (**Figure 3** and Supplementary Figure S2E): VPPV in peptides 1 and 2 (VOOV as predicted and V**P**OV); VPK in peptides 1 and 2 (VOK as predicted and V**P**K); VPV in peptide 4 (VOV as predicted and V**P**V); and CPPPPG in peptide 3 (CPOOOG as predicted and C**O**OOOG). A very high proportion of the observed motifs did not follow the extended Pro hydroxylation code for their first Pro residue.

**Table 3:** Exceptions to the proposed rules of Pro/Hyp location.

Accession number	Predicted location of Hyp residues	Observed motifs	Frequency of occurrence
At4g38770	KPOOK	KPOOK	19/29
		KP**P**OK	5/29
		K**OPP**K	4/29
		KP**PP**K	1/29
At1g09750	QOV	QOV	0/73
		Q**P**V	73/73
	VON	VON	0/21
		V**P**N	21/21
At1g31580	RPI	RPI	34/49
		R**O**I	15/49
	RPT	R**O**TRPT	23/3411/34
			
	RPR	RPR	11/34
		R**O**R	23/34
	VOG	VOG	33/55
		V**P**G	22/55
	VOI	VOI	52/55
		V**P**I	3/55
	FPOR	FPOR	55/55
		F**O**OR	55/55
	LPOY	LPOY	9/34
		LP**P**Y	23/34
At3g08030	VOS	VOS	0/21
		V**P**S	21/21
	VOF	VOF	3/63
		V**P**F	60/63
	VOH	VOH	0/20
		V**P**H	20/20
	LPL	LPL	18/21
		L**O**L	3/21
	SOL	SOL	0/48
		S**P**L	48/48
	SOG	SOG	0/49
		S**P**G	49/49
At2g10940	VOOV	VOOV	1/140
		V**P**OV	133/140
		V**PP**V	6/140
	VOK	VOK	5/119
		V**P**K	114/119
	VOV	VOV V**P**V	0/22 22/22

*Only motifs observed at most 20 times are considered in this table.*

## Discussion

This work has allowed mapping the Pro/Hyp residues in five CWPs and comparing the prediction of Pro/Hyp location according to a previously proposed extended Pro hydroxylation code (Kieliszewski and Lamport, 1994; Canut et al., 2016). Among these five proteins, only AtPRP4 was assumed to contain Hyp residues because it is known as a structural CWP with a high content of Pro residues and canonical amino acid motifs such as KKPCPP (7 occurrences) and PPV (14 occurrences) (Showalter et al., 2010). However, to our knowledge, its pattern of Pro hydroxylation has not yet been described. Regarding the other four proteins, our results have allowed enlarging the number of protein families possibly modified at the post-translational level by the hydroxylation of Pro residues. They have also shown that the Pro hydroxylation patterns can be variable at a given amino acid position.

The prediction of Hyp residue location could be done with a high level of confidence in one of the so-called HRGPs using the extended Pro hydroxylation code probably because this code was designed from the analysis of such protein sequences (Kieliszewski and Lamport, 1994; Canut et al., 2016). They include proteins rich in Pro, Ala, Ser and Thr residues such as (i) extensins with repetitive S(P)_n ≥ 2_ and YXY motifs, (ii) arabinogalactan proteins (AGPs) with AP/PA/SP/TP repeats and Pro-rich proteins (PRPs) with PPVX[KT], KKPCPP and PPV motifs (Showalter et al., 2010) or chimeric proteins containing a Pro-rich domain with XP_n_Y motifs (Hijazi et al., 2012; Canut et al., 2016). However, although prediction of the location of Pro/Hyp residues in the AtPRP4 sequence was very efficient (94.9% of successful predictions), some variability could be observed at a low frequency, particularly in the KPPPK motif, with 19 canonical Pro hydroxylation patterns (KPOOK) out of the 29 observed patterns and 10 variants (KPPOK, KOPPK, and KPPPK).

For the other proteins rich in Pro residues, many exceptions to the extended Pro hydroxylation code could be observed. In particular, in At1g31580, only about one third of the three amino acid-motifs had Hyp at the predicted location in RPI/T/R and VPG/I motifs and the FPPR motifs was systematically found with two Hyp residues (55/55). Besides, only one predicted VOOV motif could be recorded in At2g10940 out of the 140 observed peptides. The major variant was VPOV (133/140). A similar situation was found for the predicted VOK and VOV motifs (5/119 and 0/22 observations, respectively). For proteins having a low content in Pro residues, the prediction of Pro/Hyp location was also inefficient. Only a few Hyp residues could be observed and at a low frequency. Unexpected Hyp residues were also found in a previous study focused on class III peroxidases (Nguyen-Kim et al., 2016). For these proteins, a few Hyp residues were observed in CPN/Q/R, DPA, GPS/N, HPD, IPD, and LPA/Q/S motifs whereas some Pro residues were observed in APF/A, VPT, SPT/D, and TPG/L motifs. These results suggest that the extended Pro hydroxylation code cannot be used for such proteins. They also show that the rule established for the hydroxylation of the Pro residue within the EPA motif of sporamin cannot be applied (Shimizu et al., 2005). Based on mutagenesis of the surrounding amino acids, it was shown that the hydroxylation of the Pro residue required the following environment in tobacco BY-2 cells: [AVSTG]-P-[AVSTGA]-[GAVPSTC]-[APSDE].

Our results raise the question of the specificity of prolyl-4 hydroxylases (P4Hs) which have to recognize some features on the target protein at the level of its primary amino acid sequence or the secondary/tertiary structure. The specificity of three out of the 13 P4Hs of *A. thaliana* was characterized. P4H1 was shown to preferentially hydroxylate the second Pro residue in PPG motifs (Hieta and Myllyharju, 2002). All the peptides hydroxylated by P4H2 have at least three consecutive Pro residues and the third of them is preferentially hydroxylated (Tiainen et al., 2005). Finally, P4H5 was shown to hydroxylate Pro residues in SP_4_ motifs in a sequential way, but never on the fourth Pro residue (Velasquez et al., 2015). Besides, P4H2 and P4H13 were assumed to complement the Pro hydroxylation pattern of SP_4_ motifs in extensins (Velasquez et al., 2015). The characterization of additional P4Hs will give clues to understand this process which is probably tightly regulated because of its importance for biological activity. Indeed, this PTM is the first step prior to *O*-glycosylation: (i) poly-arabinosylation in extensins; or (ii) complex *O*-glycans like type II arabinogalactans (AGs), type III AGs or peanut agglutinin (PNA) AGs in AGPs, allergens or AtAGP31, respectively (Hijazi et al., 2014). None of the five CWPs analyzed in this work is known to be *O*-glycosylated. However, a previous proteomic study based on affinity chromatography with PNA, a lectin specific for galactose residues, has allowed identifying a protein of the same family as At3g08030 (Zhang et al., 2011). The next step will consist in correlating the presence of Hyp residues to *O*-glycosylation. Finally, the presence of Pro/Hyp peptide variants raises the question of the role of Hyp residues as previously discussed for class III peroxidases (Nguyen-Kim et al., 2016). This variability could be incidental or contribute to the regulation of the biological activity of CWPs.

Altogether, Pro hydroxylation events are probably more abundant in CWPs than initially thought, but the precise rules of this PTM need additional experiments to be fully described. Some clues can be proposed from our results. For example, a Hyp residue is found in the VPX motifs of AtPRP4 (77 Pro hydroxylations among the 82 observed VPX motifs) and At1g31580 (85 out of 110), whereas it is mainly a Pro residue in the three other proteins (only 15 Pro hydroxylations out of the 406 observed VPX motifs). The Pro residues in the 97 observed SPX motifs of At3g08030 were never hydroxylated whereas only a few lack of Pro hydroxylation have been described in cell wall Pro-rich proteins (Canut et al., 2016). A systematic mining of MS data is now required to permit the identification of other CWPs or secreted peptides containing Hyp residues and to map them. This task is challenging because, as mentioned above, 17.5% of the CWPs identified in one of our rosette experiments had at least one peptide carrying a Hyp residue. However, the amount of MS data corresponding to all these proteins was not sufficient to perform a relevant statistical analysis and to propose yet a further expanded Pro hydroxylation code. Finally, such a code should probably take into account tissue-specific patterns as for *O*-glycosylation (Estevez et al., 2006) and protein families. This work paves the way for a better description of Pro hydroxylation patterns in CWPs.

## Materials and Methods

### Extraction of Proteins from Cell Walls

*Arabidopsis thaliana* plants were cultivated in growth chambers at 22°C with a photoperiod of 16 h light/8 h dark. Rosettes and mature stems were collected after 4 and 6 weeks, respectively. The detailed description of the experiments is given in our previous articles (Hervé et al., 2016; Duruflé et al., 2017). Three biological replicates were performed for each experiment. Briefly, cell walls were purified as described (Feiz et al., 2006). Proteins were extracted from lyophilized cell walls in four steps using a 5 mM acetate buffer pH 4.6 complemented with 0.2 M CaCl_2_ (two successive extractions) or 2 M LiCl (*ditto*) (Irshad et al., 2008). The four protein extracts were combined prior to further analysis.

### Analysis of Proteins by LC/MS-MS and Bioinformatics

The same amount of each protein extract (40 μg) was analyzed by LC-MS/MS. In the case of rosettes, two types of analysis were performed: (i) the first one after separation of proteins by a short 1D-electrophoresis in three fractions prior to *in gelo* tryptic digestion, (ii) the second one by shotgun analysis of the extracted proteins after tryptic digestion (Hervé et al., 2016). In the case of stems, only the second method was used (Duruflé et al., 2017). LC-MS/MS analyses were performed with a Q-exactive instrument (Thermo Fisher Scientific, Villebon-sur-Yvette, France) as described (Feiz et al., 2006; Hervé et al., 2016). All the MS/MS data were made publicly available in the PROTICdb^1^ and *WallProtDB* databases^2^. The following modifications were taken into account for peptide identification: Met oxidation, Pro hydroxylation, N-ter acetylation, N-ter deamidation of Glu, N-ter deamidation of Cys and loss of H_2_O on N-ter Glu. The lists of peptides allowing the identification of the five CWPs studied in detail in this article are given in Supplementary Table S1. The search for *N*-glycosylation motifs was performed with PROSITE^3^.

## Author Contributions

HD and VH performed the protein extractions from purified cell walls and contributed to the analyses of results. TB and MZ did the MS/MS analyses. CD and EJ initiated the research, designed the study and discussed the results. EJ coordinated the analysis of the results and the writing of the manuscript. All authors read and approved the final manuscript.

## Conflict of Interest Statement

The authors declare that the research was conducted in the absence of any commercial or financial relationships that could be construed as a potential conflict of interest.

## Footnotes

1 http://proteus.moulon.inra.fr/w2dpage/proticdb/angular/

2 http://polebio.lrsv.ups-tlse.fr/WallProtDB/

3 http://prosite.expasy.org/
