# Supplementary material for: Proline Hydroxylation in Cell Wall Proteins: Is It Yet Possible to Define Rules?
Source: Front Plant Sci. 2017 Oct 17;8:1802. doi: 10.3389/fpls.2017.01802 (PMC5651053; doi:10.3389/fpls.2017.01802)
Supplement: Supplementary file 3 [file Supplementary_Figure_S1.PDF]

Supplementary Figure S1

Duruflé, Hervé *et al.*

Hydroxylation of Pro residues in five cell wall proteins as shown by MS/MS data (see Supplementary Table S1): At4g38770, At1g09750, At1g31580, At3g08030, At2g10940

Data from Hervé, Duruflé *et al.* (2016) Proteomics 16: 3183-3187 (rosettes Col0, experiments 1 and 2)
Duruflé *et al.* (2017) Proteomics (doi: 10.1002/pmic.201600449) (stems Col0)
Access to data in *WallProtDB* (www.polebio.lrsv.ups-tlse.fr/WallProtDB/)

For each protein, the predicted locations of Pro and Hyp residues are on the left, and the observed peptide variants are framed on the right. The predicted signal peptide is in light blue. Underlined Pro and Hyp residues are unexpected ones according to the extended Pro hydroxylation code. The frequency of occurrence of each peptide is indicated between brackets. Numbers corresponding to rosettes experiment 1, experiment 2 and stems are in violet, red and blue respectively. Numbers inside stars are the same between predicted peptides (on the left) and observed peptide variants (on the right).

At4g38770 (AtPRP4)

Legend	
purple arrow	Rosettes Col0 – experiment 1
red arrow	Rosettes Col0 – experiment 2
blue arrow	Stems Col0

MRILPEPRGSVPCLLLLVSVLLSATLSLA
RVVEVVGYAESKIKTOHAFSGLRVTIDCKVNKGHFVTKGSGNIDDK
GKFGLNIPHDIVSDNGALKEECYAQLHSAAGTOCPAHDGLESTKIV
FLSKSGDKHILGLKQNLKFSOEICVSKFFWPMPKLPOFK

1 GFDHPFPLPOOLELPOFLK (3/4,6/6,4/4)
KPCPOK
2 YSOOVEVOOOVOVYEPOOKK (4/7,5/6,10/11)
EIPOOVOVYDPOOKK (1/1)
3 EVOOOVOVYKPOOK (0/3)
VELPOOIPK (3/3)
KPCPOKPOK
4 IEHPOOVOVYKPOOK (5/5,1/1,1/4)
5 IEKPOOVOVYKPOOK (0/2,0/1,1/3)
IEHPOOVOVHK (4/4,3/3)
LPKKPCPOK
6 KVDPOOVOVHKPOTK (5/6,3/3,7/7)
KPCPOK
7 KVDPOOVOVHKPOOK (3/5,1/2,1/3)
IVIPOOK
4 IEHPOOVOVYKPOOK
IEHPOIYIPOIVK (8/8,6/6,8/8)
8 KPCPOOVOIYKPOVVIPK (3/4,1/1,4/6)
KPCPOOVOVYKPOVVVIPK (2/2,1/1,1/1)
KPCPPLPQLPOLPK
FPOLPOK
YIHHPKFGKWPPLPOHP

1 GFDHPFOLPOOLELPOFLK (1/4)

2 YSOOVEVOOOVOVYEPOPKK (3/7)
YSOOVEVOOOVOVYEOOOKK (1/6,1/11)

3 EVPOOVOVYKPOOK (3/3)

4 IEHOPOVOVYKPOOK (2/4)
IEHOPOVOVYKPPOK (1/4)

5 IEKPOOVOVYKOPPK (2/2,1/3)
IEKOPOVOVYKPPOK (1/1)
IEKPOOVPVYKPOOK (1/3)

6 KVDPOOVOVHKOPTK (1/6)

7 KVDPOOVOVHKOPPK (1/5)
KVDPOOVOVHKPPOK (1/5,2/3)
KVDPOOVOVHKPPPK (1/2)

8 KPCPPPVPIYKPPVVIPK (1/4)
KOCPOOVOIYKPOVVIPK (2/6)

At1g09750 (Asp protease)

➡	Rosettes Col0 – experiment 1
➡	Rosettes Col0 – experiment 2
➡	Stems Col0

MASSSLHFFFFLTLLLPFTFT
TATR
1 DTCATAAODGSDDLSIIPINAK (0/1,0/1)
CSOFAOTHVSASVIDTVLHMASSDSHR
LTYLSSLVAGKPK
PTSVOVASGNQLHIGNYVVRAK
LGTPOQLMFMVLDTSNDAVWLPCSGCSGCSNASTSFNTNSSSTYST
VSCSTAQCTQAR
GLTCPSSSOQOSVCSFNQSYGGDSSFSASLVQDTLTLAODVIPNFS
FGCINSASGNSLPOQGLMGLGR
GPMSLVSQTTSLYSGVFSYCLPSFR (41/41,24/24,22/22)
SFYFSGSLK
2 LGLLGQOK (0/3,0/1,0/1)
SIR
YTOLLRNPR
RPSLYYVNLTGVSVGSVQVOVDPVYLTFDANSGAGTIIDSGTVITR
3 FAQOVYEAIRDEFRK (0/34,0/19,0/20)
QVNVSSFSTLGAFDTCFSADNENVAOK
ITLHMTSLDLK
LPMENTLIHSSAGTLTCLSMAGIR (33/33,16/16,14/14)
QNANAVLNVIANLQQNLR
4 ILFDVONSR (0/11,0/6,0/4)
5 IGIAOEPCN (0/2,0/1)

DTCATAAPDGSDDLSIIPINAK (1/1,1/1) 1

LGLLGQPK (3/3,1/1,1/1) 2

FAQPVYEAIRDEFRK (34/34,19/19,20/20) 3

ILFDVPNSR (11/11,6/6,4/4) 4

IGIAPEPCN (2/2,1/1) 5

At1g31580 (ECS1, CXc750, possibly involved in defense mechanisms)

Rosettes Col0 – experiment 1

MASSIVSSMFLFLLLLLVFPHIDNVLG
ARMELR
1 ELGEINYADPLGFTR
2 PIVOIHVOGFPOR (0/54)
3 RPTIPQLPOYRPR (2/34)
RCPFCYPOOOOK
AFPKNSOSH

1
(GFTR) OIVOIHVOGFOOR (9/49)
(GFTR) OIVOIHVPGFOOR (3/49)
(GFTR) OIVPIHVOGFOOR (3/49)
(GFTR) PIVOIHVOGFOOR (16/49)
(GFTR) PIVOIHVPGFOOR (18/49)

2
IVOIHVPGFOOR (1/6)
IVOIHVOGFOOR (5/6)

3
RPTIOQLPOYRPR (9/34)
ROTIOQLPPYROR (23/34)

At3g08030 (DUF642)

- ➡ (purple) Rosettes Col0 – experiment 1
- ➡ (red) Rosettes Col0 – experiment 2
- ➡ (blue) Stems Col0

MAVPKAIILPILLLICGAALG
1 AOASEGYLR (0/10)
2 NGNFEESOK (0/8)
KTDMKK
TVLLGK
3 NALPEWETTGFVEYIAGGPQOGGMYFPVAHGVHAVR (1/7)
LGNEATISQK
LEVKPGSLYALTFGASR (24/24,12/12,9/9)
TCAQDEVLR
4 VSVOSQSGDLPLQTLYNSFGGDVYAWAFVAK (0/13,0/3,0/5)
TSQVTVTFHNPGVQEDPACGPLLDAVAIK (12/12,6/6,6/6)
ELVHPIYTR (10/10,6/6,6/6)
GNLVK
5 NGGFEEGPHR (10/12)
6 LVNSTQGVLLPOK (0/1)
7 QEDLTSOLPGWIIESLK (0/19,0/13,0/16)
AVK
8 FIDSKYFNVOFGHAAIELVAGK (3/36,0/13,0/14)
ESAIAQVIR
9 TSOGQTYTLSFVVGDAK (0/19,0/17,0/13)
NDCHGSMMVEAFAAR
10 DTLKVOHTSVGGGHVK (0/18,0/1,0/1)
TASFKFKAVEAR
TRITFFSGFYHTK
KTDTVSLCGPVIDEIVVSHVA (26/26,12/12,14/14)

1: APASEGYLR (10/10)
2: NGNFEESPK (8/8)
3: NALPEWETTGFVEYIAGGPQPGGMYFPVAHGVHAVR (6/7)
4: VSVPSQSGDLPLQTLYNSFGGDVYAWAFVAK (10/13,3/3,5/5); VSVPSQSGDLOLQTLYNSFGGDVYAWAFVAK (3/13,0/3,0/5)
5: NGGFEEGOHR (2/12)
6: LVNSTQGVLLPPK (1/1)
7: QEDLTSPLPGWIIESLK (19/19,13/13,16/16)
8: FIDSKYFNVPFGHAAIELVAGK (33/36,13/13,14/14)
9: TSPGQTYTLSFVVGDAK (19/19,17/17,13/13)
10: DTLKVPHTSVGGGHVK (18/18,1/1,1/1)

At2g10940 (homologous to non-specific lipid transfer protein)

Rosettes Col0 – experiment 1
Rosettes Col0 – experiment 2
Stems Col0

MDSSKLSSLSLCLFLICIIYLPQHSLA
CGSCNPRKGGKHSOKAOK
1 LPVOOVTVOK
1 LPVOOVTVOK (0/88,0/19,0/12)
1 LPVOOVTVOK
2 LPVOOVTIPK (0/6,0/8,0/7)
1 LPVOOVTVOK
1 LPVOOVTVOK
1 LPVOOVTVOK
1 LPVOOVTVOK
1 LPVOOVTVOK
1 LPVOOVTVOK
LPLPOISGLPIPOVVGPNLPLPPLPIVGPILPOGTTPOATGGK
3 DCPOOOGSVKPOSGGGK (1/9)
ATCPIDTLK (7/7,2/2)
LGACVDLLGGLVK
IGLGDPAVNK (6/6,4/4,1/1)
CCPLLK
GLVEVEAAACLCTTLK
4 LKALDLNLYVOVALQLLLTCGK (0/22)
NPOOGYTCSI

LPVOOVTVPK (1/88)
LPVPOVTVPK (83/88,16/19,9/12)
LPVPOVTVOK (1/88,1/19,2/12) 1
LPVPPVTVOK (1/88)
LPVPPVTVPK (2/88,2/19,1/12)

LPVPOVTIPK (6/6,8/8,7/7) 2

DCOOOOGSVKPOSGGGK (8/9) 3

ALDLNLYVPVALQLLLTCGK (22/22) 4
