# Supplementary material for: Proline Hydroxylation in Cell Wall Proteins: Is It Yet Possible to Define Rules?
Source: Front Plant Sci. 2017 Oct 17;8:1802. doi: 10.3389/fpls.2017.01802 (PMC5651053; doi:10.3389/fpls.2017.01802)
Supplement: Supplementary file 4 [file Supplementary_Figure_S2.PDF]

Supplementary Figure S2

Duruflé, Hervé *et al.*

Some examples of MS/MS spectra allowing the localization of Hyp residues in the amino acid sequence of AtPRP4 (At4g38770)

Data from Hervé, Duruflé *et al.* (2016) Proteomics 16: 3183-3187
and Duruflé et al. (2017) Proteomics
(doi: 10.1002/pmic.201600449)

Access in *WallProtDB*
(http://www.polebio.lrsv.ups-tlse.fr/WallProtDB/)

GFDHPFPLPOOLELPOFLK

Sample information

#	log(e)	log(I)	m+h	delta	ζ	sequence
9640	-5.5	6.28	2206.1587	0.0010	3/3	ppfk^{161} GFDHPFPLPPPLELPPFLK^{179}kpcp (0) —

mods: P170+Oxidation, P171+Oxidation, P176+Oxidation

20140307_albenne_103_1_E01_Col-22-1-1.mzXML scan 9640 (charge 3)

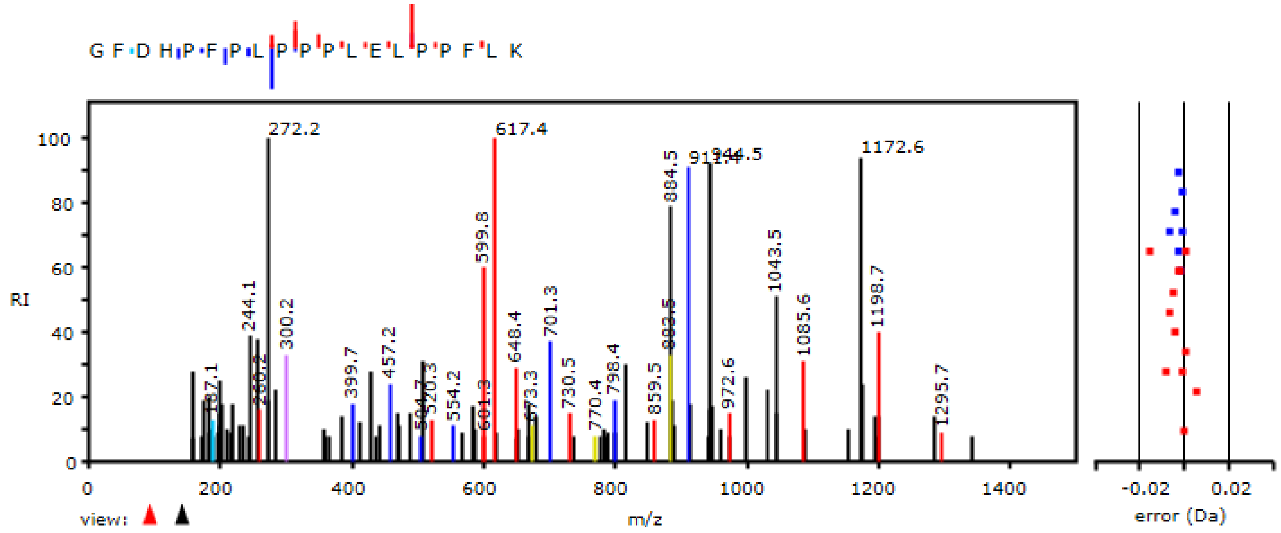

matched/total:	# ions: 24%			intensity: 31%			µ: -0.00, σ: 0.0039 Da					
**bond**	+1y	+1y-17	+1y-18	+1b	+1b-17	+1b-18	+2y	+2y-17	+2y-18	+2b	+2b-17	+2b-18
G 1	2149.137	2132.111	2131.127	58.029	41.002	40.018	1075.072	1066.559	1066.067	29.518	21.005	20.513
F 2	2002.069	1985.042	1984.058	205.097	188.071	187.087	1001.538	993.025	992.533	103.052	94.539	94.047
D 3	1887.042	1870.015	1869.031	320.124	303.098	302.114	944.025	935.511	935.019	160.566	152.052	151.560
H 4	1749.983	1732.956	1731.972	457.183	440.157	439.173	875.495	866.982	866.490	229.095	220.582	220.090
P 5	1652.930	1635.904	1634.920	554.236	537.209	536.225	826.969	818.455	817.963	277.622	269.108	268.616
F 6	1505.862	1488.835	1487.851	701.304	684.278	683.294	753.434	744.921	744.429	351.156	342.643	342.151
P 7	1408.809	1391.782	1390.798	798.357	781.331	780.347	704.908	696.395	695.903	399.682	391.169	390.677
L 8	1295.725	1278.698	1277.714	911.441	894.415	893.431	648.366	639.853	639.361	456.224	447.711	447.219
P 9	1198.672	1181.646	1180.662	1008.494	991.467	990.483	599.840	591.326	590.834	504.751	496.237	495.745
P 10	1085.624	1068.598	1067.614	1121.542	1104.515	1103.531	543.316	534.803	534.311	561.274	552.761	552.269
P 11	972.577	955.550	954.566	1234.589	1217.563	1216.579	486.792	478.279	477.787	617.798	609.285	608.793
L 12	859.493	842.466	841.482	1347.673	1330.647	1329.663	430.250	421.737	421.245	674.340	665.827	665.335
E 13	730.450	713.424	712.440	1476.716	1459.689	1458.705	365.729	357.215	356.723	738.862	730.348	729.856
L 14	617.366	600.339	599.355	1589.800	1572.773	1571.789	309.187	300.673	300.181	795.404	786.890	786.398
P 15	520.313	503.287	502.303	1686.853	1669.826	1668.842	260.660	252.147	251.655	843.930	835.417	834.925
P 16	407.266	390.239	389.255	1799.900	1782.874	1781.890	204.136	195.623	195.131	900.454	891.941	891.449
F 17	260.197	243.171	242.187	1946.969	1929.942	1928.958	130.602	122.089	121.597	973.988	965.475	964.983
L 18	147.113	130.087	129.103	2060.053	2043.026	2042.042	74.060	65.547	65.055	1030.530	1022.017	1021.525

Other observed ions:

Ion type	m/z	Ion type	m/z
a[6]	673.304	a[7]	770.357
a[8]	883.441		

Residue modification sets tested:

Complete mods:
i. Carbamidomethyl@C

Potential mods:
i. Oxidation@M, Oxidation@P
ii. Oxidation@M, Oxidation@P

N-terminal:
i. Ammonia-loss@Q, Ammonia-loss@C, Dehydrated@E (peptide)
ii. ragged, Acetyl (protein)

Rosettes Col0, experiment 1/ Data 1

GFDHPFOLPOOLELPOFLK

Sample information

#	log(e)	log(I)	m+h	delta	ζ	sequence
9543	-3.3	5.91	2222.1536	0.0083	3/3	ppfk[161] GFDHPFPLPPPLELPPFLK[179]kpcp (0) —

mods: P167+Oxidation, P170+Oxidation, P171+Oxidation, P176+Oxidation

20140307_albenne_103_1_E01_Col-22-1-1.mzXML scan 9543 (charge 3)

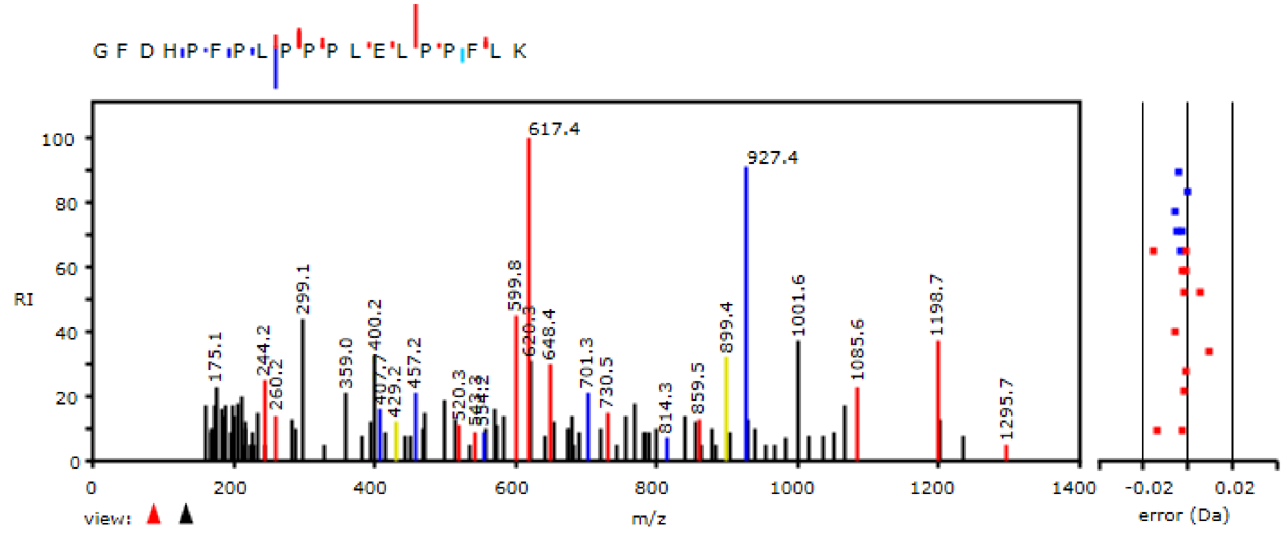

matched/total:	# ions: 20%			intensity: 35%			μ: -0.00, σ: 0.0047 Da					
**bond**	+1y	+1y-17	+1y-18	+1b	+1b-17	+1b-18	+2y	+2y-17	+2y-18	+2b	+2b-17	+2b-18
G1	2165.132	2148.105	2147.121	58.029	41.002	40.018	1083.070	1074.556	1074.064	29.518	21.005	20.513
F2	2018.064	2001.037	2000.053	205.097	188.071	187.087	1009.535	1001.022	1000.530	103.052	94.539	94.047
D3	1903.037	1886.010	1885.026	320.124	303.098	302.114	952.022	943.509	943.017	160.566	152.052	151.560
H4	1765.978	1748.951	1747.967	457.183	440.157	439.173	883.493	874.979	874.487	229.095	220.582	220.090
P5	1668.925	1651.898	1650.914	554.236	537.209	536.225	834.966	826.453	825.961	277.622	269.108	268.616
F6	1521.857	1504.830	1503.846	701.304	684.278	683.294	761.432	752.919	752.427	351.156	342.643	342.151
P7	1408.809	1391.782	1390.798	814.352	797.325	796.341	704.908	696.395	695.903	407.680	399.166	398.674
L8	1295.725	1278.698	1277.714	927.436	910.409	909.425	648.366	639.853	639.361	464.222	455.708	455.216
P9	1198.672	1181.646	1180.662	1024.489	1007.462	1006.478	599.840	591.326	590.834	512.748	504.235	503.743
P10	1085.624	1068.598	1067.614	1137.536	1120.510	1119.526	543.316	534.803	534.311	569.272	560.759	560.267
P11	972.577	955.550	954.566	1250.584	1233.558	1232.574	486.792	478.279	477.787	625.796	617.282	616.790
L12	859.493	842.466	841.482	1363.668	1346.642	1345.658	430.250	421.737	421.245	682.338	673.824	673.332
E13	730.450	713.424	712.440	1492.711	1475.684	1474.700	365.729	357.215	356.723	746.859	738.346	737.854
L14	617.366	600.339	599.355	1605.795	1588.768	1587.784	309.187	300.673	300.181	803.401	794.888	794.396
P15	520.313	503.287	502.303	1702.848	1685.821	1684.837	260.660	252.147	251.655	851.927	843.414	842.922
P16	407.266	390.239	389.255	1815.895	1798.869	1797.885	204.136	195.623	195.131	908.451	899.938	899.446
F17	260.197	243.171	242.187	1962.964	1945.937	1944.953	130.602	122.089	121.597	981.986	973.472	972.980
L18	147.113	130.087	129.103	2076.048	2059.021	2058.037	74.060	65.547	65.055	1038.528	1030.014	1029.522

Other observed ions:

Ion type	m/z	Ion type	m/z
a[4]	429.183	a[8]	899.436

Residue modification sets tested:

Complete mods:
i. Carbamidomethyl@C

Potential mods:
i. Oxidation@M, Oxidation@P
ii. Oxidation@M, Oxidation@P

N-terminal:
i. Ammonia-loss@Q, Ammonia-loss@C, Dehydrated@E (peptide)
ii. ragged, Acetyl (protein)

Rosettes Col0, experiment 1/ Data 1

YSOOVEVOOOVOVYEPOOKK

Sample information

#	log(e)	log(I)	m+h	delta	ζ	sequence
3735	-7.6	6.24	2344.1599	0.0052	3/3	cppk[186] YSPPVEVPPPVPVYEPPKK[203]eipp (0) —

mods: P188+Oxidation, P189+Oxidation, P193+Oxidation, P194+Oxidation, P195+Oxidation, P197+Oxidation, P202+Oxidation, P203+Oxidation

20140307_albenne_103_1_E03_Col-22-3-1.mzXML scan 3735 (charge 3)

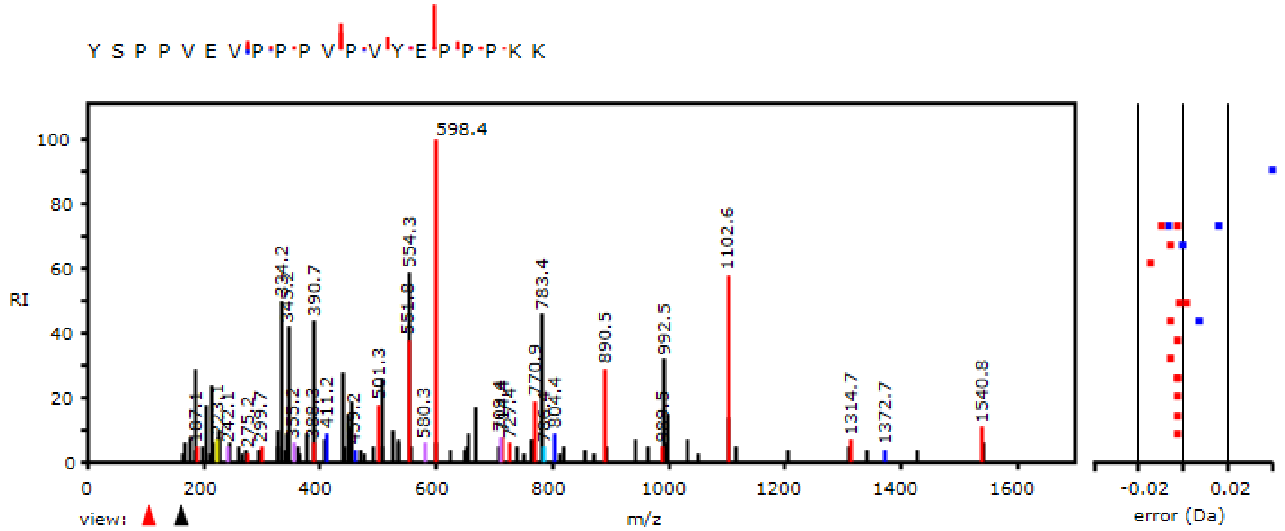

matched/total: # ions: 25% intensity: 32% μ: 0.00, σ: 0.01 Da

bond	+1y	+1y-17	+1y-18	+1b	+1b-17	+1b-18	+2y	+2y-17	+2y-18	+2b	+2b-17	+2b-18
Y1	2181.096	2164.070	2163.086	164.071	147.044	146.060	1091.052	1082.539	1082.047	82.539	74.026	73.534
S2	2094.064	2077.038	2076.054	251.103	234.076	233.092	1047.536	1039.023	1038.531	126.055	117.542	117.050
P3	1981.017	1963.990	1963.006	364.150	347.124	346.140	991.012	982.499	982.007	182.579	174.066	173.574
P4	1867.969	1850.943	1849.958	477.198	460.172	459.188	934.488	925.975	925.483	239.103	230.589	230.097
V5	1768.901	1751.874	1750.890	576.267	559.240	558.256	884.954	876.441	875.949	288.637	280.124	279.632
E6	1639.858	1622.832	1621.847	705.309	688.283	687.299	820.433	811.919	811.427	353.158	344.645	344.153
V7	1540.790	1523.763	1522.779	804.378	787.351	786.367	770.898	762.385	761.893	402.692	394.179	393.687
P8	1427.742	1410.715	1409.731	917.425	900.399	899.415	714.375	705.861	705.369	459.216	450.703	450.211
P9	1314.694	1297.668	1296.684	1030.473	1013.446	1012.462	657.851	649.338	648.845	515.740	507.227	506.735
P10	1201.647	1184.620	1183.636	1143.521	1126.494	1125.510	601.327	592.814	592.322	572.264	563.751	563.259
V11	1102.578	1085.552	1084.568	1242.589	1225.562	1224.578	551.793	543.279	542.787	621.798	613.285	612.793
P12	989.531	972.504	971.520	1355.637	1338.610	1337.626	495.269	486.756	486.264	678.322	669.809	669.317
V13	890.462	873.436	872.452	1454.705	1437.678	1436.694	445.735	437.221	436.729	727.856	719.343	718.851
Y14	727.399	710.372	709.388	1617.768	1600.742	1599.758	364.203	355.690	355.198	809.388	800.875	800.383
E15	598.356	581.330	580.346	1746.811	1729.784	1728.800	299.682	291.168	290.676	873.909	865.396	864.904
P16	501.303	484.277	483.293	1843.864	1826.837	1825.853	251.155	242.642	242.150	922.436	913.922	913.430
P17	388.256	371.229	370.245	1956.911	1939.885	1938.901	194.632	186.118	185.626	978.959	970.446	969.954
P18	275.208	258.182	257.198	2069.959	2052.933	2051.949	138.108	129.594	129.102	1035.483	1026.970	1026.478
K19	147.113	130.087	129.103	2198.054	2181.027	2180.043	74.060	65.547	65.055	1099.531	1091.017	1090.525

Other observed ions:

Ion type	m/z	Ion type	m/z
a[2]	223.103		

Residue modification sets tested:

Complete mods:
i. Carbamidomethyl@C

Potential mods:
i. Oxidation@M, Oxidation@P
ii. Oxidation@M, Oxidation@P

N-terminal:
i. Ammonia-loss@Q, Ammonia-loss@C, Dehydrated@E (peptide)
ii. ragged, Acetyl (protein)

Rosettes Col0, experiment 1/ Data 1

YSOOVEVOOOVOVYEPOPKK

Sample information

#	log(e)	log(I)	m+h	delta	ζ	sequence
3831	-6.7	6.46	2328.1650	0.0056	3/3	cppk[186] YSPPVEVPPPVPVYEPPPKK[205]eipp (0) —

mods: P188+Oxidation, P189+Oxidation, P193+Oxidation, P194+Oxidation, P195+Oxidation, P197+Oxidation, P202+Oxidation

20140307_albenne_103_1_E03_Col-22-3-1.mzXML scan 3831 (charge 3)

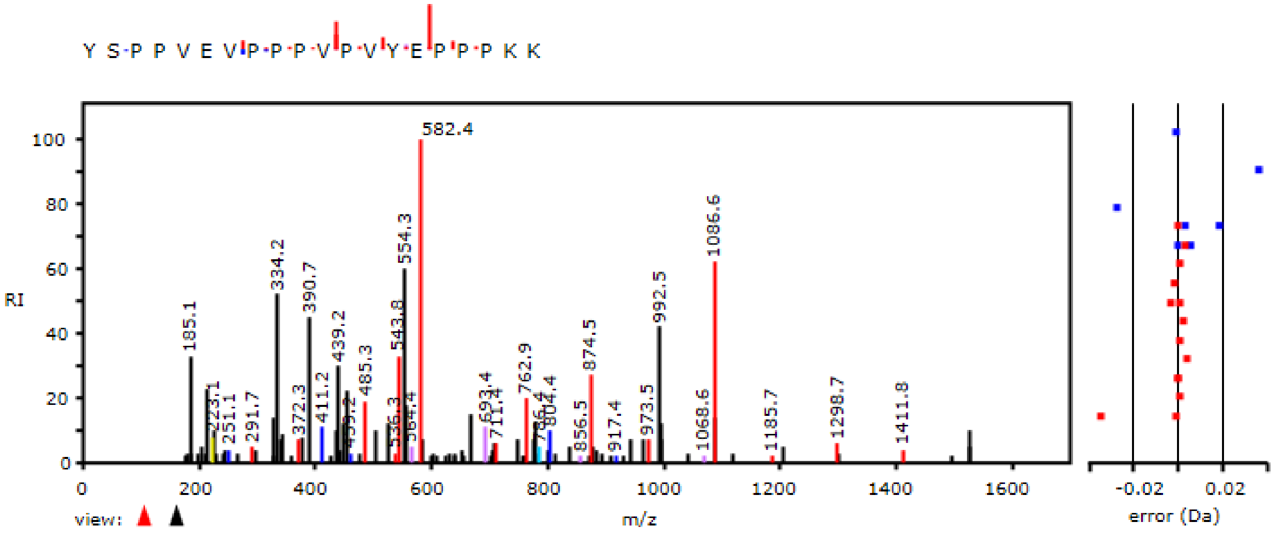

matched/total: # ions: 25% intensity: 35% μ: -0.00, σ: 0.01 Da

bond	+1y	+1y-17	+1y-18	+1b	+1b-17	+1b-18	+2y	+2y-17	+2y-18	+2b	+2b-17	+2b-18
Y1	2165.102	2148.075	2147.091	164.071	147.044	146.060	1083.054	1074.541	1074.049	82.539	74.026	73.534
S2	2078.069	2061.043	2060.059	251.103	234.076	233.092	1039.538	1031.025	1030.533	126.055	117.542	117.050
P3	1965.022	1947.995	1947.011	364.150	347.124	346.140	983.015	974.501	974.009	182.579	174.066	173.574
P4	1851.974	1834.948	1833.964	477.198	460.172	459.188	926.491	917.977	917.485	239.103	230.589	230.097
V5	1752.906	1735.879	1734.895	576.267	559.240	558.256	876.957	868.443	867.951	288.637	280.124	279.632
E6	1623.863	1606.837	1605.853	705.309	688.283	687.299	812.435	803.922	803.430	353.158	344.645	344.153
V7	1524.795	1507.768	1506.784	804.378	787.351	786.367	762.901	754.388	753.896	402.692	394.179	393.687
P8	1411.747	1394.721	1393.736	917.425	900.399	899.415	706.377	697.864	697.372	459.216	450.703	450.211
P9	1298.699	1281.673	1280.689	1030.473	1013.446	1012.462	649.853	641.340	640.848	515.740	507.227	506.735
P10	1185.652	1168.625	1167.641	1143.521	1126.494	1125.510	593.329	584.816	584.324	572.264	563.751	563.259
V11	1086.583	1069.557	1068.573	1242.589	1225.562	1224.578	543.795	535.282	534.790	621.798	613.285	612.793
P12	973.536	956.509	955.525	1355.637	1338.610	1337.626	487.271	478.758	478.266	678.322	669.809	669.317
V13	874.467	857.441	856.457	1454.705	1437.678	1436.694	437.737	429.224	428.732	727.856	719.343	718.851
Y14	711.404	694.377	693.393	1617.768	1600.742	1599.758	356.206	347.692	347.200	809.388	800.875	800.383
E15	582.361	565.335	564.351	1746.811	1729.784	1728.800	291.684	283.171	282.679	873.909	865.396	864.904
P16	485.309	468.282	467.298	1843.864	1826.837	1825.853	243.158	234.645	234.153	922.436	913.922	913.430
P17	372.261	355.234	354.250	1956.911	1939.885	1938.901	186.634	178.121	177.629	978.959	970.446	969.954
P18	275.208	258.182	257.198	2053.964	2036.938	2035.954	138.108	129.594	129.102	1027.486	1018.972	1018.480
K19	147.113	130.087	129.103	2182.059	2165.033	2164.049	74.060	65.547	65.055	1091.533	1083.020	1082.528

Other observed ions:

Ion type	m/z	Ion type	m/z
a[2]	223.103		

Residue modification sets tested:

Complete mods: i. Carbamidomethyl@C

Potential mods:
i. Oxidation@M, Oxidation@P
ii. Oxidation@M, Oxidation@P

N-terminal:
i. Ammonia-loss@Q, Ammonia-loss@C, Dehydrated@E (peptide)
ii. ragged, Acetyl (protein)

Rosettes Col0, experiment 1/ Data 1

YSOOVEVOOOVOVYEPOPKK

Sample information

#	log(e)	log(I)	m+h	delta	ζ	sequence
3739	-6.8	6.38	2328.1650	-0.0015	3/3	cppk[188] YSPPVEVPPPVPVYEPPPKK[205]eipp (0) —

mods: P188+Oxidation, P189+Oxidation, P193+Oxidation, P194+Oxidation, P195+Oxidation, P197+Oxidation, P202+Oxidation

20140307_albenne_103_1_E01_Col-22-1-1.mzXML scan 3739 (charge 3)

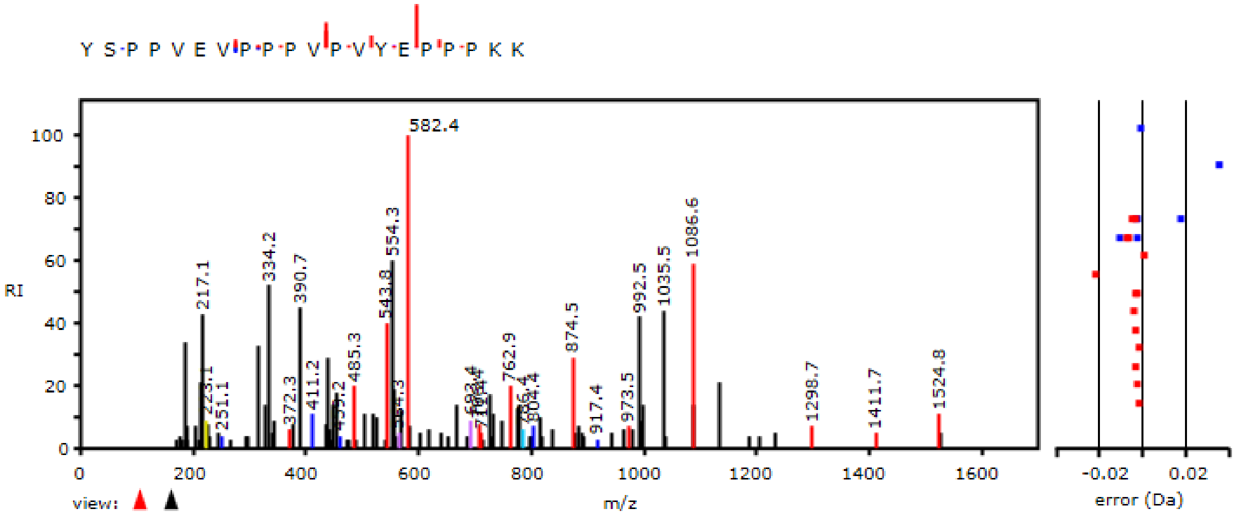

matched/total: # ions: 22% intensity: 29% µ: -0.00, σ: 0.01 Da

bond	+1 y	+1 y-17	+1 y-18	+1 b	+1 b-17	+1 b-18	+2 y	+2 y-17	+2 y-18	+2 b	+2 b-17	+2 b-18
Y1	2165.102	2148.075	2147.091	164.071	147.044	146.060	1083.054	1074.541	1074.049	82.539	74.026	73.534
S2	2078.069	2061.043	2060.059	251.103	234.076	233.092	1039.538	1031.025	1030.533	126.055	117.542	117.050
P3	1965.022	1947.995	1947.011	364.150	347.124	346.140	983.015	974.501	974.009	182.579	174.066	173.574
P4	1851.974	1834.948	1833.964	477.198	460.172	459.188	926.491	917.977	917.485	239.103	230.589	230.097
V5	1752.906	1735.879	1734.895	576.267	559.240	558.256	876.957	868.443	867.951	288.637	280.124	279.632
E6	1623.863	1606.837	1605.853	705.309	688.283	687.299	812.435	803.922	803.430	353.158	344.645	344.153
V7	1524.795	1507.768	1506.784	804.378	787.351	786.367	762.901	754.388	753.896	402.692	394.179	393.687
P8	1411.747	1394.721	1393.736	917.425	900.399	899.415	706.377	697.864	697.372	459.216	450.703	450.211
P9	1298.699	1281.673	1280.689	1030.473	1013.446	1012.462	649.853	641.340	640.848	515.740	507.227	506.735
P10	1185.652	1168.625	1167.641	1143.521	1126.494	1125.510	593.329	584.816	584.324	572.264	563.751	563.259
V11	1086.583	1069.557	1068.573	1242.589	1225.562	1224.578	543.795	535.282	534.790	621.798	613.285	612.793
P12	973.536	956.509	955.525	1355.637	1338.610	1337.626	487.271	478.758	478.266	678.322	669.809	669.317
V13	874.467	857.441	856.457	1454.705	1437.678	1436.694	437.737	429.224	428.732	727.856	719.343	718.851
Y14	711.404	694.377	693.393	1617.768	1600.742	1599.758	356.206	347.692	347.200	809.388	800.875	800.383
E15	582.361	565.335	564.351	1746.811	1729.784	1728.800	291.684	283.171	282.679	873.909	865.396	864.904
P16	485.309	468.282	467.298	1843.864	1826.837	1825.853	243.158	234.645	234.153	922.436	913.922	913.430
P17	372.261	355.234	354.250	1956.911	1939.885	1938.901	186.634	178.121	177.629	978.959	970.446	969.954
P18	275.208	258.182	257.198	2053.964	2036.938	2035.954	138.108	129.594	129.102	1027.486	1018.972	1018.480
K19	147.113	130.087	129.103	2182.059	2165.033	2164.049	74.060	65.547	65.055	1091.533	1083.020	1082.528

Other observed ions:

Ion type	m/z	Ion type	m/z
a[2]	223.103		

Residue modification sets tested:

Complete mods:
i. Carbamidomethyl@C

Potential mods:
i. Oxidation@M, Oxidation@P
ii. Oxidation@M, Oxidation@P

N-terminal:
i. Ammonia-loss@Q, Ammonia-loss@C, Dehydrated@E (peptide)
ii. ragged, Acetyl (protein)

Rosettes Col0, experiment 1/ Data 3

YSOOVEVOOOVOVYEOOOKK

Sample information

#	log(e)	log(I)	m+h	delta	ζ	sequence
6711	-3.2	6.11	2360.1548	-0.0001	3/3	cppk[186] YSPPVEVPPPVPVYEPPPKK[205]eipp (0) —

mods: P188+Oxidation, P189+Oxidation, P193+Oxidation, P194+Oxidation, P195+Oxidation, P197+Oxidation, P201+Oxidation, P202+Oxidation, P203+Oxidation

20151009_durufle_141_A01_T22_1_col.mzXML scan 6711 (charge 3)

Y S P P V E V P P P V P V Y E P P P K K

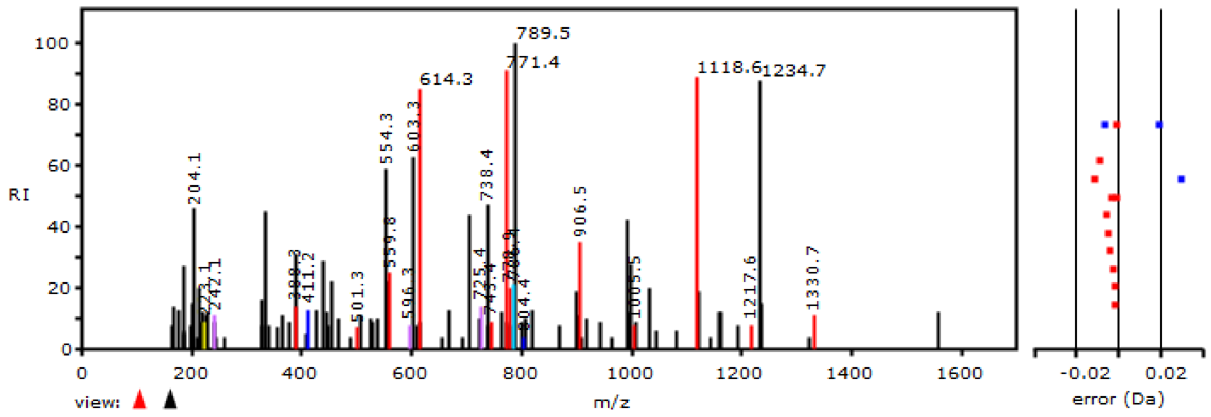

matched/total: # ions: 19% intensity: 26% μ: 0.00, σ: 0.0100 Da

bond	+1y	+1y-17	+1y-18	+1b	+1b-17	+1b-18	+2y	+2y-17	+2y-18	+2b	+2b-17	+2b-18
Y1	2197.091	2180.065	2179.081	164.071	147.044	146.060	1099.049	1090.536	1090.044	82.539	74.026	73.534
S2	2110.059	2093.033	2092.049	251.103	234.076	233.092	1055.533	1047.020	1046.528	126.055	117.542	117.050
P3	1997.012	1979.985	1979.001	364.150	347.124	346.140	999.009	990.496	990.004	182.579	174.066	173.574
P4	1883.964	1866.937	1865.953	477.198	460.172	459.188	942.486	933.972	933.480	239.103	230.589	230.097
V5	1784.896	1767.869	1766.885	576.267	559.240	558.256	892.951	884.438	883.946	288.637	280.124	279.632
E6	1655.853	1638.826	1637.842	705.309	688.283	687.299	828.430	819.917	819.425	353.158	344.645	344.153
V7	1556.785	1539.758	1538.774	804.378	787.351	786.367	778.896	770.383	769.891	402.692	394.179	393.687
P8	1443.737	1426.710	1425.726	917.425	900.399	899.415	722.372	713.859	713.367	459.216	450.703	450.211
P9	1330.689	1313.663	1312.679	1030.473	1013.446	1012.462	665.848	657.335	656.843	515.740	507.227	506.735
P10	1217.642	1200.615	1199.631	1143.521	1126.494	1125.510	609.324	600.811	600.319	572.264	563.751	563.259
V11	1118.573	1101.547	1100.563	1242.589	1225.562	1224.578	559.790	551.277	550.785	621.798	613.285	612.793
P12	1005.525	988.499	987.515	1355.637	1338.610	1337.626	503.266	494.753	494.261	678.322	669.809	669.317
V13	906.457	889.430	888.446	1454.705	1437.678	1436.694	453.732	445.219	444.727	727.856	719.343	718.851
Y14	743.394	726.367	725.383	1617.768	1600.742	1599.758	372.200	363.687	363.195	809.388	800.875	800.383
E15	614.351	597.325	596.341	1746.811	1729.784	1728.800	307.679	299.166	298.674	873.909	865.396	864.904
P16	501.303	484.277	483.293	1859.859	1842.832	1841.848	251.155	242.642	242.150	930.433	921.920	921.428
P17	388.256	371.229	370.245	1972.906	1955.880	1954.896	194.632	186.118	185.626	986.957	978.444	977.952
P18	275.208	258.182	257.198	2085.954	2068.927	2067.943	138.108	129.594	129.102	1043.481	1034.967	1034.475
K19	147.113	130.087	129.103	2214.049	2197.022	2196.038	74.060	65.547	65.055	1107.528	1099.015	1098.523

Other observed ions:

Ion type	m/z	Ion type	m/z
a[2]	223.103		

Residue modification sets tested:

**Complete mods:**	i. Carbamidomethyl@C
**Potential mods:**	i. Oxidation@M, Oxidation@P
**N-terminal:**	i. Ammonia-loss@Q, Ammonia-loss@C, Dehydrated@E (peptide)
	ii. ragged, Acetyl (protein)

Stems Col0, Data 1
